# *“A lot of medical students, their biggest fear is failing at being seen to be a functional human”:* disclosure and help-seeking decisions by medical students with health problems

**DOI:** 10.1186/s12909-021-03032-9

**Published:** 2021-12-05

**Authors:** Bar Shahaf-Oren, Ira Madan, Claire Henderson

**Affiliations:** 1 MSc programme, King’s College London, Institute of Psychiatry, Psychology and Neuroscience, London, UK; 2 Occupational Health Department, Guy’s and St Thomas’ NHS Foundation Trust and King’s College, London, UK; 3 Health Service and Population Research Department, King’s College London, Institute of Psychiatry, Psychology and Neuroscience, London, UK

**Keywords:** Disclosure, Help-seeking, Medical students, Health problems, Disabilities

## Abstract

**Background:**

Although medical students have a duty to seek advice for their health conditions, they tend to avoid disclosure and help-seeking behaviours, therefore potentially posing a risk to themselves and their patients. The literature regarding their decisions to seek help or disclose health conditions is limited. The study’s purpose was to explore the factors that determine disclosure and help-seeking decision processes by medical students who have health conditions with or without disability.

**Methods:**

We recruited by purposive sampling and conducted in-depth semi-structured interviews with 11 male and female medical students from a UK medical school, who had physical or mental health disorders. Thematic analysis was used to identify themes. A mix of inductive and deductive techniques was used while using an organising framework proposed by Llewellyn-Thomas (1995).

**Results:**

The impact of individuals’ features, such as personality traits on medical students’ disclosure and help-seeking decisions were identified. Different aspects of the condition, such as its type and severity were found to influence these decisions. Participants made an evaluation of the potential receiver of a disclosure, consisting of factors such as the receiver’s characteristics and attitudes. The culture of the medical environment, such as role models, had a major impact on their decisions. Finally, systemic factors, such as the lack of clarity of policies influenced students’ decisions.

**Conclusion:**

Medical students’ disclosure and help-seeking decision processes are influenced by risk-benefit evaluations and factors in interlinked spheres of their lives. They tend to avoid or postpone disclosure and seeking help, especially when the university is involved, due to a perceived risk to their future. Future research should examine the role of personality traits and the medical culture. Medical schools should encourage earlier help-seeking and disclosure behaviours by clarifying procedures and building trust via online and confidential platforms; interpersonal channels and normalisation processes within the medical education and the profession as a whole.

**Supplementary Information:**

The online version contains supplementary material available at 10.1186/s12909-021-03032-9.

## Introduction

Medical students are at high risk of mental health disorders relative to the general population. The literature consistently recognises the high prevalence of anxiety, depressive disorders and suicidal ideation [1, 2], addictions [3], stress and burnout [4–6], headaches and fatigue [7]. Less is known about medical students’ physical health [8], although one longitudinal study demonstrated that this can deteriorate while at medical school [9]. Widespread changes in laws and attitudes towards discrimination of people with disabilities [10–12] have widened access to higher education and the medical profession [13, 14]. In the United Kingdom, the General Medical Council promotes the inclusion of individuals with disabilities or health problems in the medical profession, and emphasises medical schools’ responsibility for taking care of such students [15, 16]. The students, in turn, have the duty to acknowledge, disclose and seek advice for their conditions [14].

Yet, a growing body of literature recognises the lack of seeking mental and physical health care among medical students as a widespread problem [9, 17]. Medical students are usually resistant towards help-seeking and treatment [4, 18], unwilling to disclose mental health problems or declare disabilities [14, 19], and prefer to use non-formal ways to receive support by informally consult their friends, family, peers or colleagues [8]. Similar issues have been noted among qualified doctors who also have a high prevalence of mental health disorders [20], lack of, or delay in accessing health-care [21], self-diagnosis, self-prescribing and harmful behaviours [22]. These constitute a risk for the doctors themselves, for their patients and the public as a whole [21, 23]. Doctors and medical students have been found to have similar barriers to access care such as fear of consequences, confidentiality and stigma [17, 24]. Respondents who describe a fear of stigma may be most likely to fear its behavioural manifestation i.e. discrimination. However, negative experiences might also result from the knowledge and attitudinal components, such as lack of understanding and avoidance respectively [25]. The evidence suggests that poor health-care attitudes form during medical education [8, 19, 24], influenced by socialisation processes and role models [4, 26]. Therefore a comprehensive understanding of health-care attitudes and the development of interventions to facilitate disclosure and accessing health care are crucial for a medical student’s future career and their patients alike [21, 27].

Literature about decision-making processes involves various fields and perspectives [28] Although the literature holds several models in the area of help-seeking and disclosure decision processes [29, 30] they are limited in their ability to inform the design and analysis of this study. Llewellyn-Thomas [31] reflects a holistic approach by viewing patients’ health-care decision-making as unique and, therefore, as one that should include various perspectives and interactive elements that occur on several layers. This framework is also suggested to be the basis of the development of Decision Aids (Das), designed to help people make informed and personal choices [32]. Llewellyn-Thomas’s *Intrapersonal “Rubik’s cube”* organises the basic processes involved in an individual’s decision-making about health-care and includes different elements, presented on each of the Rubik’s cube’s faces which represents the complexity and interaction of the phenomena. The first face consists of four elements of the decision problem regarding health care: health status, treatment processes, time periods and decision-making participation. The second face represents the individual’s perspectives towards the decisions: *Information* a person holds regarding their health condition; *Expectations* refers to the person’s assessments of the decision possibilities e.g. risks or benefits of care *Preference* refers to the degree of willingness or satisfaction of the individual with aspects of the decision. The third face represents the person’s characteristics, personality, socio-demographics and clinical features. The *interpersonal sphere* considers the impact of other important and relevant players to the process. The *extra-personal sphere* reflects the socio-political context of the decision. By taking into account different layers, this framework presents a holistic approach towards the understanding of disclosure and help-seeking decisions.

Guided by this framework, this study was done with the ultimate goal of developing an online intervention to support medical students’ decision making about help-seeking and disclosure. The question proposed in the current study is: what are the factors that determine medical students’ disclosure and help-seeking decision-making processes? Therefore, the first aim was to explore how medical students make decisions about disclosure, help-seeking and get advice on the potential impact of their condition on their fitness to study, and/or future practice. The second aim is to identify their perceived barriers to disclosure, help-seeking and getting advice.

## Methods

### Setting

A large UK medical school, with over 400 medical students per year.

### Participants

Undergraduate medical students who had health condition or disability, either self-determined or diagnosed by a doctor.

### Sample and recruitment

We recruited by purposive sampling. A maximum variation sampling was used to reach diversity in terms of age, academic year, gender and ethnicity. All medical students were invited to participate via a newsletter and social media, providing the Participant Information Sheet, study details and purpose. Participants were informed about the voluntary nature of the study and their right to withdraw at any point and that non-participation would not lead to any consequences.

The interview topic guide (see Additional file 1) focused on different aspects of the participants’ disclosure and help-seeking behaviours such as how they dealt with these decisions, when they took actions and the people involved. Interviews were recorded and transcribed verbatim with the participants’ consent. Semi-structured interviews were conducted from March to June 2017 by one of the researchers (BO).

### Analysis

The transcripts were transferred to NVivo software and analysed thematically using Braun and Clarkes’ [33] six stages as a guide. A mix of inductive and deductive techniques was used to enable meanings to arise from the data as well as top down processes when using Llewellyn-Thomas [31] recommendations and framework to organise the data. The first transcript was coded by three researchers to ensure common understanding and improve quality of analysis. Another interview was coded by two of the researchers and the rest was done by one.

## Results

Of the 11 participants, 6 were men and 5 were women, ranging in age from 19 to 26 (Mean age 23). Participants had at least one health condition, mental health problem, or both. Physical conditions included severe and/or chronic illnesses. Mental disorders included a range of common mental disorders (Table 1 summarises the demographic characteristics of the participants- Additional file 2). Interviews lasted between 23 and 82 min (average of 51).

**Table 1:** Demographic and educational characteristics of participants (*n* = 11)

		Number of students
Gender	Male	6
	Female	5
Age	Mean age: 23.09	11
	Range: 19–26	
Ethnicity	White-British	8
	Asian	2
	African	1
Year of study	Year 1	2
	Year 2	3
	Year 3	5
	Year 5	1
Type of condition	Physical condition	3
	Mental condition	3
	Comorbid mental conditions	2
	Physical and mental conditions (including comorbid)	3
Additional education	Intercalation (optional additional year of study)	4
	Bachelors of Science	3
	Masters of Science	1
	Interruptions (repeated the year)	6

**Fig. 1:**
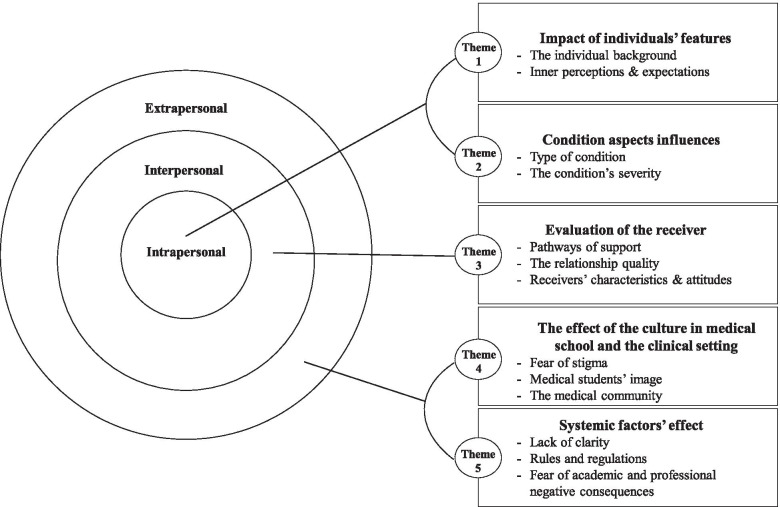
Interlinked spheres diagram

Thematic analysis of the interviews generated five main themes that were organised in three interlinked spheres (**in bold)**: intrapersonal, interpersonal and extra-personal, consistent with Llewellyn-Thomas [31] recommendation (Please see Fig. 1 for illustration- Additional file 3).

Each theme addresses both study aims, i.e. how the students make disclosure and help-seeking decisions as well as their barriers to do so. It is important to acknowledge that some quotes may reflect several interlinked themes. For example, past experiences from previous access to support, may reflect both intra and extrapersonal spheres, due to their interlinked nature. We chose to present the quotes according to where we believed to be most suitable and clarifying. The main themes are highlighted in ***italics bold.*** Participants’ quotes are highlighted in *italics*.

### Intrapersonal sphere

#### Impact of individuals’ features

The participant’s individual background consisted of several aspects: Many talked about personality traits such as the degree of openness that influenced their preferences in terms of their decisions. Some talked about the impact of them “being private” on non-disclosure while others noted disclosure as part of their openness.

Past experiences in childhood or adolescence were also mentioned as associated with difficulties to disclose:

*“I think there are things that you don’t want to tell […] especially if you have been in counselling when you are a young teenager, there is definitely a limit on what you can say to them before they will report you […]”.*

A few participants talked about their culture as another influential factor:

*“It is a very difficult thing to bring up […] because, I guess, culturally, it is not something that is spoken about".*

Participants’ inner perceptions and expectations have also been identified as influential features. Many expressed their lack of initial understanding of their condition: *“In truth, I probably didn’t even know it myself. I knew I wasn’t the same that I had been...”* and the role of insight as vital for, and the basis of, these decisions: *“If you don’t have much insight, it is really hard to disclose and ask for help”*.

Most of them spoke about perceived risks from sharing or seeking help for their condition, comprising time consumption; fear of escalating the situation; being judged; being exposed and fear of treatment side effects.

In contrast, only a few, mostly with mental health problems, mentioned perceived intrapersonal benefits and included gaining a better understanding of their condition; decreased feelings of guilt increased feelings of hope and well-being, for example: *“It allowed me to be less hard on myself”.*

#### Influences of aspects of the condition

Participants evaluated many aspects of the condition when making decisions. They mostly voiced greater difficulties regarding mental health problems compared with physical conditions, including those participants with both types of conditions: *“It is not as comfortable as saying, ‘I broke my leg, or, ‘I got the flu’, for some reason”.*

They identified several explanations for this, including lack of treatment availabilities and limited inner comprehensibility:

*“With mental illness, sometimes you are worried that there just isn’t really a treatment for it […]. So am I cursed or something? […]. It is more complex and unclear”*.

Moreover, several students with physical conditions mentioned that it is harder to disclose physical conditions that are more intimate in nature: *“I feel like it is different talking about (condition Y); it feels more intrusive and uncomfortable”*.

Participants also compared visible versus invisible conditions. They voiced their ability to choose whether they wanted to disclose an invisible condition: *“from looking at me […] people wouldn’t know that there is anything wrong”.*

in contrast to visible conditions that cannot be hidden: “*I feel like I have to explain myself more anyway with the (visible condition). It is obvious when I have operations […]”*.

Another influential factor for deciding to disclose or seek help for the majority of the students was the condition’s severity:

*“It really was just me being at my lowest point […] I just can’t continue this way […] I just felt like I needed help desperately”*.

### Interpersonal sphere

#### Evaluation of the receiver

##### Pathways of support

The students spoke about the relevant “players” on informal or formal pathways of support who were associated with different perceived risks and benefits that influenced their decision processes.

Informal pathways included the participants’ family; partners; friends within or outside the university; housemates; university-related peer groups, online apps and telephone hot-lines.

The perceived risks from informal sources were getting attention that they did not want; being labelled or being a burden: *“I didn’t tell (my family) too much about what was going on […] I didn’t really want to, effectively, burden them with worrying about me”*.

The benefits included receiving academic materials from peers; receiving others’ support and feeling they are not alone when meeting someone who is dealing with a similar condition: *“I spoke to a friend and she told me how last year she went through the same thing. […] it normalises the whole thing”.*

Formal pathways of support consisted of non-university or university sources. Non-university sources refer both to private and public systems such as General Practitioners (prinary care physicians), counsellors, psychiatrists and specialists the students usually used for advice or referral letters for university.

The risks of using non-university sources, especially by students with mental health problems, included receiving a harmful or an ineffective treatment and hospitalisation: *“A GP is slightly more likely to institutionalise me […] I just wouldn’t want to tell them because it would just make things more difficult”*. Perceived benefits included receiving beneficial care and advice; confidentiality and formal support: *“I just really wanted someone to talk to that wasn’t a friend or family; I wanted someone outside”.*

University sources include administrative and academic staff, such as personal tutors and clinical advisors (responsible for the students’ pastoral care and academic progress). Participants usually used these sources only for necessary academic situations, such as missing exams or multiple absences: *“I told them only when it became necessary, like if I was missing things”.*

Students tended to disclose the minimum information required:

*"With official (university) channels, I have tended to try to give less information”.*

The students mentioned risks such as unhelpful guidance and lack of response; negative consequences due to staff being part of the medical school: *“If I am telling her (clinical advisor) something, I am telling the school”* and being seen differently: *“The risk-benefit of me telling her (personal tutor) versus not telling her was that she might look at me a bit differently if she knew”.*

Perceived benefits included academic flexibility and useful referrals and information: *“They (the staff) do know a lot about what you should expect, and they do tell you that”.*

##### Relationship quality

An evaluation of the relationship quality was made regarding both formal and informal potential receivers of a disclosure and most commonly consisted of closeness with the receiver: *“For me, one of the deciding factors is how close I am to that person. I think that definitely dictates whether I will tell them”*.

Many students indicated that lack of acquaintance with the potential receiver in formal pathways could lead them to be less willing to disclose or seek help: *“The staff, you don’t know them well at all […] so I didn’t want to be very open about everything because it is quite hard to talk about”.*

Further, many students, especially those with mental health problems, expressed that they were likely to share and seek help from people with similar conditions due to a mutual understanding:

*"There was a girl who was on my course, actually she shared first that she had (a mental health) disorder. […] people just stared at her. […] and then later I went up to her and was like, ‘I actually have (it) myself, so I understand what you are going through'. She actually did advise me to seek help […]".*

##### The potential receiver’s characteristics and attitudes

Participants commonly expressed the importance of sensitive and non-judgmental attitudes of the receivers: *“I shared it with (two teachers) because they were very accessible and friendly”*.

They were less likely to seek help from people considered to be critical or were difficult to talk to: *“I have a friend who is actually a medic and he just doesn’t get mental health. […] I wouldn’t talk to him about it”*.

When evaluating people from formal pathways, participants were less likely to disclose and seek-help from potential receivers whose medical speciality was one they perceived does not require a sensitive approach: *“I wouldn’t have told her (clinical advisor) about anything. She was not approachable. In a lot of ways, she was your stereotypical histopathologic. […] They don’t really see patients”*.

Alternatively, people from professions that require in-depth understanding and warmth were more likely to trigger these behaviours. The following example can reflect an evaluation of a potential receiver:

*"I feel a connection with her because she teaches medical humanities. […]. So I feel like she would be very sensitive and good. It is about her as a person rather than the role she has and what she could do for me"*.

### Extra-personal sphere

#### The effect of the culture in medical school and the clinical setting

The fear of stigma was noted as a barrier to disclose and seek help amongst the students with mental conditions: *“It is difficult for me to share because of the stigma I believe people associate with it”*.

When referring to university, most students indicated that they did not experience a direct incident of being stigmatised, but tended to interpret it as a result of them being lucky or using selective disclosure: *“I, personally, haven’t been stigmatised for it, but I have been very careful who I have told”*.

Some of them, however, have mentioned that the feeling of stigma resulted from indirect incidents, such as other students’ experiences or informal discussions: *“Even doctors, actually, in the hospital, will just sort of say, ‘Oh, psychiatry, everyone’s mad,’ and just be a bit stigmatising […]”*.

Most of them also voiced the fear of not fitting the medical students’ image, hence, “being excellent”, healthy and “strong” and by disclosing or asking for help they can be seen as less successful, weak or incapable: *“A lot of medical students, their biggest fear is failing in something, not necessarily in exams […] but failing at being seen to be a functional human”*.

Being part of the medical community was another influential factor. Several students did not know anyone else from university with similar conditions and asserted that medical students do not usually talk about their problems, which made help-seeking and disclosure decisions more difficult: *“Maybe just to know that you are not alone, with regards to disclosing and sharing […] it is something that would maybe allow someone to take the first step”*.

Still, several students acknowledged the school’s efforts to increase awareness about wellbeing and to decrease stigma, alongside the difficulties to apply the changes:

*"There are a lot of things that are changing in medicine as to how we treat patients that is not replicated as to how we are treated […] it is the way that the processes/institutions/organisations are built"*.

#### Systemic factors’ effects

Most of the students talked about the lack of clarity of policies and procedures in the medical school and in clinical settings. This includes the complexity of the referral system and the lack of information initially: *“I felt like I had to do a lot of chasing to find the right person […] I was like, ‘This is so difficult’”*.

The students also reported a lack of information about counselling services, long waiting lists and the limited number of sessions as barriers for receiving help: *“It takes ages to get an initial appointment […] It is an absolute nightmare”*. Conversely, two out of the three students that did manage to use counselling services mentioned it positively.

Another aspect that impacted their decisions was the ambiguity of the consequences:

*"There wasn’t any information on the website or any concrete things saying that, if you disclose, it is not going to affect […] your place at university. […] I was really scared about actually disclosing"*.

Rules and regulations were also commonly voiced as a systemic factor. Some of the students discussed the medical school’s demanding atmosphere, lack of flexibility and the focus on attendance: *“The atmosphere is of you have to be in as much as you can or it is going to be a problem”*.

When it came to university as a system, participants tended to avoid disclosure or postpone it to a critical point. They commonly noted their fear of negative consequences, which sometimes resulted in their withholding information:

*"Occupational Health did call me and asked me loads of questions about (my condition), and I realised, as I was answering those questions, I was lying a bit and I was downplaying it […] I have worked really hard to get into this school, and I don’t want this to get in the way of it"*.

This was often associated with a broader perspective of fitness to practice.^1^ Most of the students indicated they would not seek advice because they believed they were capable of evaluating themselves: *“I would make that decision myself and be able to evaluate that myself”*, or would rather do it through informal or private sources: *“It would not be through formal procedures because the formal procedures are not supportive”*.

They mostly explained their avoidance of seeking advice for fitness to practice due to their concerns about losing their place in medical school and their future career:

*"You have spent years working towards getting into it. […] where your main drive is to become a doctor [...] I think a lot of people have difficulties if it were to be taken away. […] that doesn’t help people getting help […]"*.

## Discussion

The students usually referred to disclosure as part of their decision processes of whether, when, how and from whom to seek help. This may further support the idea that disclosure constitutes a step towards help-seeking behaviour [34] and is useful in understanding the students’ decision-processes.

Consistent with Llewellyn-Thomas [31] framework, participants referred to various aspects of their decisions, which occurred in different spheres that influence one another. An integration of multi-sphere disclosure and help-seeking decisional factors, therefore, may be critical in developing beneficial strategies. The students were less likely to disclose and seek help unless they had to due to the deterioration and the severity of their condition, as has been found in other studies of this group [2, 17]. We also found that participants who seemed to have a more open attitude in general were more likely to disclose and seek help for their condition. This may suggest that personality traits play a crucial role in these decision processes. In accordance with another study [35] which indicated positive relationships between openness and beneficial coping strategies and negative correlations with coping avoidance, we also identified several factors that influenced students’ openness to interventions. The students may have perceived the risks (e.g. time consumption) as too high and/or been insufficiently persuaded by potential benefits such as academic performance until they had no other choice. In addition, a lack of understanding of the condition prevented participants from initiating disclosure and help-seeking decisions as has been noted before [36, 37] Finally, stigma and consequent unwillingness to admit their conditions were identified as a barrier, consistent with other studies that found that the degree of self-stigma and self-acceptance are central factors to disclosure decision-processes [36, 38, 39]. Stigma may also play a role in the decision not to disclose concealable conditions [19, 29]. In general, the students used informal pathways and avoided university officials to receive emotional support and advice in order to prevent jeopardising their academic and professional future. In case they had an academic necessity, they mostly used non-university sources to receive professional treatment and ensure confidentiality [24].

In contrast with earlier findings that students are likely to disclose to family and friends from medical school [17], students in this study were less likely to disclose and seek help from friends from medical school as compared to other friends. Regarding their families, especially for those with mental health problems, these behaviours depended on the degree of intimacy [29, 40]. Closeness was also a consideration for potential receivers from formal sources, along with perceptions about whether the receiver is sensitive and non-judgemental, or likely to be, based on their professional specialty. Concerns about not meeting a perceived medical student’s image when dealing with physical or mental conditions were also found as barriers [17, 27]. Personal and indirect incidents with peer groups and role models in the medical community and the academically demanding nature of medical education may lead medical students to feel the need to “prove” they are capable and therefore avoid these help-seeking and disclosure behaviours [41].

Students’ perceptions that disclosure or help-seeking from the university system might threaten their academic and professional future may reflect the combination of strict rules with the lack of clarity regarding consequences. The underlying choice in this context is that between their health and their future. A lack of information and clarity about the consequences reflects the lack of a single solution to health conditions, and may reflect constructive ambiguity, i.e. a deliberate approach on the part of the medical school to avoid commitments or allow flexibility [13, 42]. Finally, even when making a decision to seek help from university pathways, the complex referral systems were described as major barriers [17].

## Limitations

The research was carried out at one medical school in the UK and the results may not be externally generalisable [43]. This study was explorative and a maximum variation sampling was used to reach a diverse range of participants. However, those willing to take part in an in depth interview may represent a selected sample only partially generalisable to the study setting. For example, the disclosure reported were often between female students or to a female member of academic staff. The question whether it is easier to disclose to women should be addressed using quantitative methods. The sample included participants with a range of physical and mental health disorders. However, none of them had other types of impairments such as learning disabilities, hearing or visual impairments. This might be due to the time constraints and/or the requirement of the participants to share sensitive information. Strategies to ensure the anonymity of the participants were used at all times. Future research is needed to examine participants with other types of conditions.

Face-to-face interviews were used to gain insight of participants’ experiences and useful to create rapport and note facial expressions [44]. However, interviews rely on the responders’ ability to accurately and honestly recall experiences. The interviewer’s attitude may have impacted the interviewee’s responses [45]. The study took place in the university setting which might have prevented or motived participants to discuss certain experiences. The researcher was external to the medical school, which might have helped to increase objectivity. This might be due to the time constraints and/or the requirement of the participants to share sensitive information. Strategies to ensure the anonymity of the participants were used at all times.

## Conclusions

This study contributes to the understanding of influential factors on disclosure and help-seeking decision-processes amongst medical students with mental and physical health conditions. A larger quantitative study would be needed to determine the generalisability of these findings within the study setting, while qualitative studies at other medical schools would be needed to determine whether the findings are transferrable. Future research is needed to gain a better understanding of the influence of the medical students’ personality traits, decision tendencies and background on disclosure and help-seeking decisions. Studies should also focus on the role of the medical school’s organisational culture. An organisational culture promoting diversity and inclusion and non-discrimination policies have been noted before as an enabler of disclosure-decision making [36, 41]. This study raises the need to conduct further investigation of the impact of the medical culture on medical students’ barriers to inform organisational change. Further research is needed to examine how meeting role models or peers with similar conditions may lead to normalisation and openness to disclose and seek help, along with reassurance that disclosure rarely results in termination of studies.

This study emphasises the importance for medical students of early, positive and accessible resources to increase willingness to disclose and seek help. One way might be the development of anonymous platforms to give students useful information, including on the potential benefits of disclosure and seeking help, and hence make decisions, the most obvious delivery method being an online one to support interactivity.

## Supplementary Information

**Additional file 1.** Questioner topic guide.

**Additional file 2: Table 1**. Demographic and educational characteristics of participants (*n* = 11).

**Additional file 3: Figure 1**: Interlinked spheres diagram.

## Data Availability

The datasets generated and analysed during the current study are not available, as access to these qualitative data could lead to identification of participants. However, for reasonable request please contact C.H (author) at claire.1.henderson@kcl.ac.uk

## Footnotes

1 A statement of policy approved by the GMC that deals with the skills, knowledge, character and health to practice the medical profession safely and effectively
