# Supplementary material for: “A lot of medical students, their biggest fear is failing at being seen to be a functional human”: disclosure and help-seeking decisions by medical students with health problems
Source: BMC Med Educ. 2021 Dec 5;21:599. doi: 10.1186/s12909-021-03032-9 (PMC8645095; doi:10.1186/s12909-021-03032-9)
Supplement: Supplementary file 1 — Additional file 1. Questioner topic guide. [file 12909_2021_3032_MOESM1_ESM.docx]

Appendix A: Questioner topic guide

Participant number:

General questions:

- Year in studies:
- Are you a UK or overseas student?
- Where do you live now?
- How old are you?
- How do you describe your study experience?
- Can you describe your health problem?
- Do you consider that you have any disabilities? How does it affect your day to day life?

Interview:

- Do you think your health status affects your studies? And if so, how?
- Did you/would you tell others about your health problem or disability?
- If so, at what point did you/would you tell someone about your health problem or
  disability?
- Who would you/did you share and why?
- How would you describe your health problem/disability to those you decide to share with? What would you agree to share?
- Have you ever considered seeking for help or treatment? If so, who did you/would
  you approach?
- What will drive you to seek help? Or at what point do you think you will / you did
  consider to seek help?
- What would you/did you share?
- Who would you avoid asking for help? Why?
- Would you seek advice on your fitness to practice?
- If so, whom would you approach? And in what stage/point?
- If not, why wouldn't you?
- What if anything makes these decisions difficult?
- Do you feel you had enough information to make these decisions?
- Are there things you wish you had known earlier on about telling others or getting
  help or advice on your condition?
- Do you feel you had enough time to make these decisions?
- What else might help you to make these decisions?
