# Supplementary material for: “A lot of medical students, their biggest fear is failing at being seen to be a functional human”: disclosure and help-seeking decisions by medical students with health problems
Source: BMC Med Educ. 2021 Dec 5;21:599. doi: 10.1186/s12909-021-03032-9 (PMC8645095; doi:10.1186/s12909-021-03032-9)
Supplement: Supplementary file 2 — Additional file 2: Table 1. Demographic and educational characteristics of participants (n = 11). [file 12909_2021_3032_MOESM2_ESM.docx]

Appendix B: **Table 1. Demographic and educational characteristics of participants (n=11)**

|  |  | Number of students |
| --- | --- | --- |
| Gender | Male | 6 |
|  | Female | 5 |
| Age | Mean age: 23 | 11 |
|  | Range: 19-26 |  |
| Ethnicity | White-British | 8 |
|  | Asian | 2 |
|  | African | 1 |
| Year of study | Year 1 | 2 |
|  | Year 2 | 3 |
|  | Year 3 | 5 |
|  | Year 5 | 1 |
| Type of condition | Physical condition | 3 |
|  | Mental condition | 3 |
|  | Comorbid mental conditions | 2 |
|  | Physical and mental conditions (including comorbid) | 3 |
| Additional education | Intercalation (optional additional year of study) | 4 |
|  | Bachelors of Science | 3 |
|  | Masters of Science | 1 |
|  | Interruptions (repeated the year) | 6 |
