# Supplementary figures and images for: “A lot of medical students, their biggest fear is failing at being seen to be a functional human”: disclosure and help-seeking decisions by medical students with health problems
Source: BMC Med Educ. 2021 Dec 5;21:599. doi: 10.1186/s12909-021-03032-9 (PMC8645095; doi:10.1186/s12909-021-03032-9)

Appendix C: Figure 1: Interlinked spheres diagram


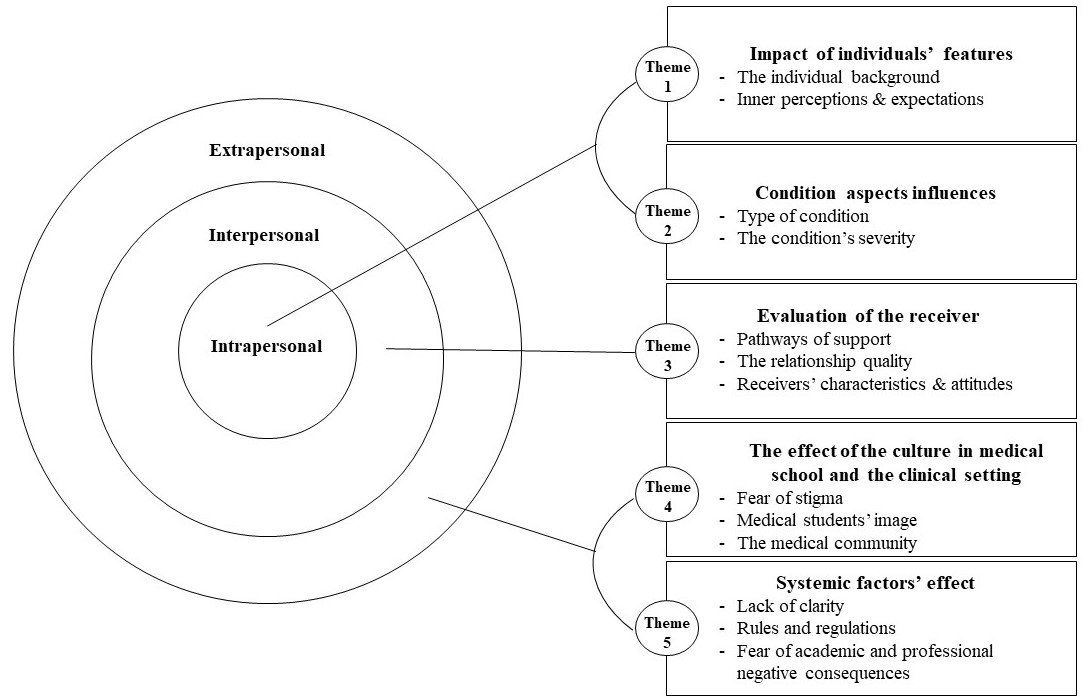

Supplement: Supplementary file 3 — Additional file 3: Figure 1: Interlinked spheres diagram. [file 12909_2021_3032_MOESM3_ESM.docx]
